# Vascular adhesion protein-1 in hemodialysis and hemodiafiltration patients: effect of single hemodialysis session on its level in regard to type of anticoagulant

**DOI:** 10.1007/s11255-016-1497-3

**Published:** 2017-01-02

**Authors:** Jolanta Malyszko, Ewa Koc-Zorawska, Piotr Kozminski, Jacek S. Malyszko

**Affiliations:** 1 2nd Department of Nephrology and Hypertension with Dialysis Unit, Medical University, M. Sklodowskiej-Curie 24a, 15-276 Białystok, Poland; 2 Dialysis Unit, Mlawa, Poland; 3 1st Department Nephrology and Transplantology with Dialysis Unit, Medical University, Białystok, Poland

**Keywords:** Hemodialysis, Hemodiafiltration, VAP-1, Anticoagulation, UFH, LMWH

## Abstract

**Purpose:**

Traditional anticoagulants used in intermittent hemodialysis (HD) are unfractionated heparin (UFH) and increasingly low molecular weight heparins (LMWHs). Repeated and prolonged exposure to UFH and/or LMWHs may further disturb hemostasis in uremic patients. Vascular adhesion protein-1 (VAP-1) is secreted by vascular smooth muscle cells, adipocytes and endothelial cells with functional monoamine oxidase activity and is elevated in atherosclerosis, diabetes mellitus and obesity. The aim of this study was to assess the effects of UFH and LMWHs on VAP-1 concentration in HD patients. The effects on single HD session on VAP-1 were also evaluated as well as VAP-1 levels in regard to type of renal replacement therapy.

**Methods:**

We studied 82 hemodialyzed patients (mean age 63 years, dialysis vintage 59 months) and 17 patients treated by means of hemodiafiltration (HDF) (mean age 59 years, HD vintage 84 months, HDF 7 months). Patients were anticoagulated with enoxaparin (*n* = 46), dalteparin (*n* = 10), nadroparin (*n* = 6) or UFH (*n* = 20) during their HD sessions. VAP-1 was assessed using kits from BioVendor, Modrice, Czech Republic.

**Results:**

Patients on HDF had significantly lower VAP-1 when compared with HD patients. We found that VAP-1 concentration in patients dialyzed by using LMWH or UFH was similar. There was no effect on HD session on VAP-1 concentration. Diabetic patients had higher serum VAP-1 than non-diabetic.

**Conclusions:**

HDF is associated with lower VAP-1 levels indicating less pronounced endothelial cell injury than hemodialysis. Type of heparin seems to have no effect on VAP-1 levels in hemodialyzed patients. However, the cross-sectional but not prospective design is a limitation of this study.

## Introduction

Vascular adhesion protein-1 (VAP-1) is a copper-containing semi-carbazide-sensitive amine oxidase (SSAO) secreted by vascular smooth muscle cells, adipocytes and endothelial cells with functional monoamine oxidase activity [1]. On the other hand, endothelial VAP-1 can act as an adhesion molecule [2] and is involved in leukocyte rolling, adhesion and transmigration, which are central steps during leukocyte extravasation to sites of inflammation, such as atherosclerotic lesions [3]. Hemodiafiltration (HDF) is a combination of convective and diffusive processes for solute removal, i.e., a combination of intermittent hemofiltration with simultaneous hemodialysis. Convection favors the elimination of higher molecular weight substances, whereas low molecular weight substances are removed from the blood by diffusion. Klingel et al. [4] have reported that HDF in comparison with high-flux HD was associated with increased procoagulant activity in the extracorporeal circuit. We have shown previously that patients on HD showed evidence of a higher degree of endothelial dysfunction than HDF subjects [5]. It appears that HD procedures induce stimulation and damage of endothelial cells and that long-term, recurrent HD treatment may predispose to vascular disorders [6]. Up to authors’ knowledge, there are no data on the VAP-1 levels in HDF, and in addition there are no reports on effects of heparin on VAP-1 in patients maintained on chronic HD. Taking all these data into consideration, the aim of the study was to assess VAP-1 levels in regard to type of renal replacement therapy, as well as the effects on HD session on VAP-1 levels in regard to three types of low molecular weight heparin (LMWH) and unfractionated heparin (UFH).

## Materials and methods

The study was performed on 82 patients with end-stage renal failure treated by means of chronic HD and 17 by HDF. Inclusion criteria were: a stable clinical state, no thrombosis or inflammation, (C-reactive protein within normal range, below 6 mg/dL according to the central laboratory cutoff), absence of acute cardiovascular complications (including uncontrolled hypertension, recent myocardial infarction within 30 days, etc.), no oral contraception in women of child-bearing age, stable and no more than twice of the normal GOT and GPT activities. None of the patients investigated had received blood transfusions for at least 1.5 months, and no drugs known to affect hemostasis were administered for at least 2 weeks prior to the study. Patients were selected from the group of HD subjects who had been receiving enoxaparin (*n* = 46), dalteparin (*n* = 10), nadroparin (*n* = 6) or UFH (*n* = 20) as an anticoagulant during their HD sessions. At the beginning of HD, enoxaparin (Clexane, Sanofi-Aventis, Paris, France), dalteparin (Fragmin, Pfizer Europe, Sandwich, Kent, UK) or nadroparin (Fraxiparie, GlaxoSmithKline, Brentford, Middlesex, UK) were administered as a single bolus of 40 mg (20–80 mg), 5000 IU (2500–7500 IU) or 3800 IU anti-Xa IU (2850–5700 anti-Xa IU), respectively, into the predialyzer arterial line. The effective dose of enoxaparin, dalteparin or nadroparin was 50 ± 15 mg (1 mg = 100 IU of anti-factor Xa activity), 5000 ± 1291 IU or 4050 ± 1090 anti-Xa IU, respectively. The dose was established on the basis of clinical guidelines: no visible fibrin clots in the arterial and venous bubble traps during HD, no clotted filters after HD and no bleeding from the fistula puncture sites after compression [7, 8] and was similar to previously reported [7–9]. UFH (Heparinum, Polfa, Warsaw, Poland) was administered as a loading dose of 2500 IU (1500–4000 IU) into the arterial line prior to the onset of HD session, followed by a continuous infusion of 3500 (2500–4500) IU via a syringe pump during HD. The infusion was stopped 30 min before the scheduled end of HD session. The effective dose of UFH was established individually by titration during the first three HD sessions. It was based on the whole-blood activated partial thromboplastin time, which was about twofold prolonged at both 30 and 120 min after the onset of HD relative to the baseline. For HDF population only enoxaparin was used.

In all dialyzed patients, blood was drawn in the morning between 8.00 a.m. and 9.00 a.m. before administration of anticoagulant and after HD/HDF from the arterial line of HD system immediately before discontinuation of the extracorporeal circulation (only for urea concentration necessary for Kt/V determination). Blood was taken without stasis. Venous blood samples were collected into 3.8% sodium citrate in 9:1 volume ratio. Samples were aliquoted and stored at −80 °C before assay. All subjects gave informed consent, and the protocol was approved by the Medical University Ethics Committee. VAP-1 was assessed using kits from BioVendor, Modrice, Czech Republic. Data given were analyzed using Statistica 10.0 computer software (Tulsa, OK, USA). Normality of variable distribution was tested using Shapiro–Wilk *W* test. Measurements normally distributed are reported as mean ± SD. ANOVA or Mann–Whitney rank-sum *U* test or Student’s *t* test was used in statistical analysis to compare differences between groups with *p* < 0.05 considered statistically significant, when appropriate. Multiple regression analysis was used to determine independent factors affecting dependent variable. Factors showing linear correlation with VAP-1 (*p* < 0.1) were included in the analysis. Adjustments for the dialysis modality were performed.

## Results

Basic clinical and biochemical characteristics of the studied patients are given in Table 1. Patients on HDF had significantly lower VAP-1 when compared with HD patients (Table 1). Patients’ hypertension (*n* = 82) had higher VAP-1 levels when compared with patients with normotension (335.30 ± 104.04 vs. 256.94 ± 129.12 ng/mL, *p* < 0.05). Patients with coronary artery disease (*n* = 67) had higher VAP-1 than patients without coronary artery disease (310.57 ± 172.16 vs. 239.34 ± 129.08 ng/mL, *p* < 0.05). Patients with residual renal function (*n* = 36) had lower VAP-1 than patients without residual renal function (243.03 ± 113.45 vs. 319.28 ± 139.73 ng/mL, *p* < 0.05). Diabetic patients (*n* = 24) had higher VAP-1 than non-diabetic (361.54 ± 123.86 vs. 291.43 ± 118.76 ng/mL, *p* < 0.05). The data were the same for HD and HDF group; therefore, they are presented as a whole group.

**Table 1:** Clinical and biochemical characteristics of studied groups

	HD (*n* = 82)	HDF (*n* = 17)
Age (years)	63 ± 17	59 ± 14
Time of renal replacement therapy (months)	59 ± 35	HD 84 ± 50 HDF 7 ± 2
Residual renal function (mL/24 h)	400 (0;1500)	200 (0;800)
KT/V	1.18 ± 0.24	1.28 ± 0.16*
Iron (μg/dL)	68.92 ± 24.65	115.64 ± 47.43**
TSAT (%)	24.57 ± 8.75	35.67 ± 9.43*
Ferritin (ng/mL)	415 (179;697)	527 (274;811)
Hemoglobin (g/dL)	11.63 ± 1.42	11.32 ± 1.35
EPO dose (U/week)	4111 ± 2778	3782 ± 2643
Total cholesterol (mg/dL)	178.65 ± 49.65	151.65 ± 50.74*
Total protein (g/dL)	6.52 ± 0.71	6.59 ± 0.55
Albumin (g/dL)	3.91 ± 0.41	3.84 ± 0.48
Total calcium (mmol/L)	2.31 ± 0.29	2.23 ± 0.34
Phosphates (mg/dL)	6.04 ± 2.39	5.79 ± 2.89
Ejection fraction (EF) (%)	59.18 ± 10.48	57. 55 ± 8.19
VAP-1 (ng/mL)	326.11 ± 119.60	240.66 ± 109.22*
Diabetes (%)	24	24
Hypertension (%)	83	82
Coronary artery disease (%)	67	71

* *p* < 0.05, ** *p* < 0.01 HD versus HDF

In univariate analysis, VAP-1 correlated with presence of diabetes (*r* = 0.29, *p* < 0.05), presence of hypertension (*r* = 0.23, *p* < 0.05), ejection fraction (*r* = −0.43, *p* < 0.01), cholesterol (*r* = −0.23, *p* < 0.05), serum iron (*r* = −0.23, *p* < 0.05), ferritin (*r* = −0.28, *p* < 0.05). In multiple regression analysis, VAP-1 was predicted in 44% by serum ejection fraction (beta value 0.36, *p* = 0.014), and ferritin (beta value 0.25, *p* = 0.039) *F* = 4.33, SE = 137.89 and *p* < 0.001.

**Fig. 1:**
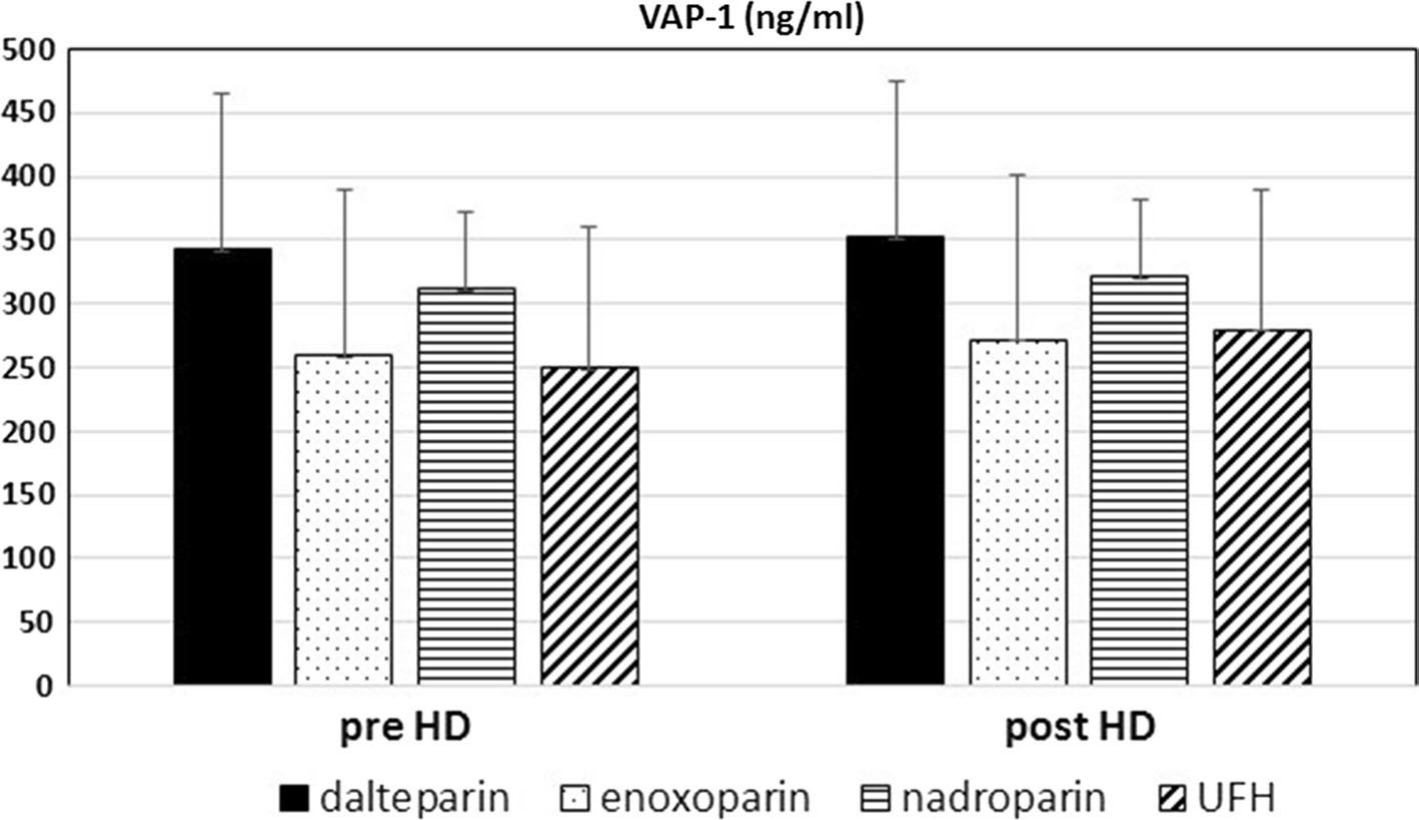
Effects of single dialysis session and different heparins on VAP-1 in hemodialyzed patients

We found that VAP-1 concentration in patients dialyzed by using enoxaparin, fraxiparine, dalteparin or UFH was similar (Fig. 1).

## Discussion

In our study we compared two dialysis modalities: HD and HDF. Chronic HDF is a relatively simple option of renal replacement therapy that may offer significant benefits for many patients who have end-stage renal disease and deserve closer consideration for these patients. As HDF favors the elimination of higher molecular weight substances, we assessed VAP-1 in HDF patients. In this paper we showed for the first time that VAP-1 levels were significantly lower in HDF when compared to HD, similarly to our previous study on renalase [10]. Moreover, we could show the effect of residual renal function on VAP-1 levels in larger group of patients than we presented previously [11]. Lin et al. [12] reported that VAP-1 levels were positively associated with the urinary albumin-to-creatinine ratio and inversely correlated with estimated GFR, suggesting that serum VAP-1 might be excreted by the kidneys. These data support our findings. Moreover, in kidney transplant recipients VAP-1 correlated with kidney function and endothelial damage as well [13]. We observed that patients with coronary artery disease had higher VAP-1 levels than patients without coronary artery disease. We did not see the effect in the smaller group [11]. Serum VAP-1 can independently predict 10-year all-cause mortality, cardiovascular mortality, in subjects with type 2 diabetes [14]. In addition, in heart transplant recipients VAP-1 was related to heart function and kidney function [15], which corroborates with presented data. In prospective cohort study (the FINRISK 2002) on 2775 participants (mean age, 60 years) followed up of 9 years, 265 participants underwent a major adverse cardiovascular event (MACE), and these participants had higher levels of VAP-1 than those without MACE (868 and 824 ng/mL, respectively, *p* < 0.001) [16]. Aalto et al. [16] concluded that VAP-1 independently predicted incident MACE (*p* = 0.0046) and MACE mortality (*p* = 0.026) in general population over 50 years of age. They also suggested that VAP-1 may be a potential new biomarker for cardiovascular diseases. Ferritin was one of the predictors of VAP-1 in our population. Being the acute-phase reactant, not only the iron storage protein [17], ferritin, paralleled VAP-1 levels in diabetic dialyzed patients [18].

As endothelial dysfunction in common in chronic kidney disease [19] and endothelial VAP-1 can participate in inflammation by binding granulocytes, lymphocytes and monocytes, with the aid of SSAO activity [1, 2], VAP-1 might be considered as a novel biomarker for cardiovascular diseases in kidney patients, in particular those with diabetes.

The new and interesting points of the study are the possible effects of the type of dialysis and the type of heparin on VAP-1 levels. Up to the authors’ knowledge, there are no data comparing dalteparin, nadroparin and enoxaparin with UFH in regard to VAP-1. We reported for the first time the effects of different heparins on VAP-1 in HD patients, but we found that VAP-1 seemed to be unaffected by dialysis session. VAP-1 is a dual-function protein and can participate in the development and progress of atherosclerosis [20]; however, linking the degree of endothelial dysfunction with use of anticoagulants with their long-term consequences needs prospective studies. As in the recent study, Lavainne et al. [21] indicated that heparin-based HD induced a major release of sFlt1, a potent inhibitor of vascular endothelial growth factor, which may exacerbate the anti-angiogenic state and thus endothelial dysfunction, commonly found in dialysis patients. In our previous study, we reported that LMWH influences thrombin-activatable fibrinolysis inhibitor (TAFI) and other hemostatic parameters in hemodialyzed patients to a lesser degree than UFH [22]. Long-term use of anticoagulants in HD patients may thus influence endothelial function.

Our study has several limitations due to its cross-sectional design, which makes it difficult to determine the causality between serum VAP-1 and HDF and type of anticoagulation. The small sample size and enrollment in such an ethnically homogeneous Caucasian population limit the results to be generalized, but the study was performed on HD and HDF population which could be an advantage. The majority of studies in renal replacement therapy are designed for HD patients, and sometimes it is difficult to extrapolate the data into other modality of dialysis. The small sample in regard to HDF population is due to the fact that this modality is not reimbursed, and thus, the difference in costs between HD and HDF is covered by dialysis unit which limits the possibility to offer HDF to more patients. We should bear in mind that choosing the best dialysis modality and type of anticoagulation depends not only on the clinical status but also on price and healthcare policy.

In conclusion, HDF is associated with lower VAP-1 levels indicating less pronounced endothelial cell injury favoring thus type of treatment. Type of heparin seems to have no effect on VAP-1 levels in HD patients. However, the cross-sectional but not prospective design is a limitation of this study.
